# Implications of navigation in thoracolumbar pedicle screw placement on screw accuracy and screw diameter/pedicle width ratio

**DOI:** 10.1016/j.bas.2023.101780

**Published:** 2023-07-11

**Authors:** Eric Mandelka, Jula Gierse, Felix Zimmermann, Paul A. Gruetzner, Jochen Franke, Sven Y. Vetter

**Affiliations:** Research group Medical Imaging and Navigation in Trauma and Orthopedic Surgery (MINTOS), Department of Orthopedics and Trauma Surgery, BG Klinik Ludwigshafen, Ludwig-Guttmann-Str. 13, 67071, Ludwigshafen, Germany

**Keywords:** Spinal navigation, Intraoperative imaging, Image-guided surgery, C-Arm navigation, Screw accuracy, Screw size

## Abstract

**Introduction:**

There is ample evidence that higher accuracy can be achieved in thoracolumbar pedicle screw placement by using spinal navigation. Still, to date, the evidence regarding the influence of the use of navigation on the screw diameter to pedicle width ratio remains limited.

**Research question:**

The aim of this study was to investigate the implications of navigation in thoracolumbar pedicle screw placement not only on screw accuracy, but on the screw diameter to pedicle width ratio as well.

**Material and methods:**

In this single-center single-surgeon study, 45 Patients undergoing navigated thoracolumbar pedicle screw placement were prospectively included. The results were compared with a matched comparison group of patients in which screw placement was performed under fluoroscopic guidance. The screw accuracy and the screw diameter to pedicle width ratio of every screw were compared between the groups.

**Results:**

Screw accuracy was significantly higher in the navigation group compared to the fluoroscopic guidance group, alongside with a significant increase of the screw diameter to pedicle width ratio by approximately 10%. In addition, both the intraoperative radiation dose and the operating time tended to be lower in the study group.

**Conclusion:**

This study was able to show that navigated thoracolumbar pedicle screw placement not only increases the accuracy of screw placement but also facilitates the selection of the adequate screw sizes, which according to the literature has positive effects on fixation strength. Meanwhile, the use of navigation did not negatively affect the time needed for surgery or the patient's intraoperative exposure to radiation.

## Highlights

• Matched comparison of navigated and fluoroscopy-guided pedicle screw placement.
• Accuracy was significantly higher with navigation compared to fluoroscopic guidance.
• In both groups, none of the breaches resulted in postoperative morbidities.
• Screw diameter to pedicle width ratio was significantly higher using navigation.
• No significant differences found for operating time and patient radiation dose.

## 1 Introduction

The use of pedicle screws for dorsal stabilization in various indications is a well-established surgical technique (Solitro et al., 2019; Rosinski et al., 2021; Miller et al., 2016; Verma et al., 2016a). However, serious adverse events like neurovascular injury, CSF leakage or visceral injury due to misplaced screws always remain a concern (Miller et al., 2016). In addition, long-term complications, such as screw loosening, screw-rod disconnections, or bent/broken screws as well as adjacent segment degeneration (ASD) have been reported (Solitro et al., 2022; Lonstein et al., 1999; Vaccaro et al., 1995; Bianco et al., 2017).

In recent years, there has been a growing interest in the use of modern technology based to improve the accuracy of pedicle screw placement in minimally invasive spine surgery, such as 3D imaging-based navigation and robotic assistance (Miller et al., 2016; Du et al., 2022; Luther et al., 2015; Scarone et al., 2018; Perdomo-Pantoja et al., 2019; Shin et al., 2012). The availability of a variety of 3D-imaging devices and navigation systems for spinal navigation has led to an increasing number of studies evaluating their image quality, radiation dose, accuracy, and usability (Miller et al., 2016; Luther et al., 2015; Scarone et al., 2018; Farah et al., 2018; Hecht et al., 2018; Beisemann et al., 2022). Accordingly, there is ample evidence that navigated techniques outperform non-navigated techniques in terms of accuracy (Perdomo-Pantoja et al., 2019; Shin et al., 2012; Fichtner et al., 2018; Sun et al., 2020; Tarawneh et al., 2021; Verma et al., 2016b). Several authors have identified the selection of adequately sized screws as key factor for optimal fixation strength (Bianco et al., 2017; Misenhimer et al., 1989; Shea et al., 2014). While the risk for pedicle perforation and its associated complications increases with the use of larger screws, the literature suggests that a higher screw diameter to pedicle width (SD/PW) ratio may provide biomechanical advantages such as increased stability and load-bearing capacity (Solitro et al., 2019, 2022; Matsukawa et al., 2021). Still, to date, the evidence regarding the influence of the use of navigation on the SD/PW ratio remains limited (Luther et al., 2015).

The aim of this study was to investigate the implications of navigation in thoracolumbar pedicle screw placement not only on pedicle screw accuracy, but on the SD/PW ratio as well.

## 2 Material and Methods

In this study, we prospectively collected data for all procedures of thoracolumbar pedicle screw placement using intraoperative 3D imaging guided navigation performed by a single senior spine surgeon in our institution between August 2021 and Mai 2022. The study was approved by the ethics committee responsible (application number 2021–16061). All patients gave informed consent, and the procedures were in compliance with the 1964 Helsinki Declaration and its subsequent amendments.

The results were compared with a control group of fluoroscopy-guided procedures performed in our institution performed in our institution between 2018 and 2022. To allow for reasonable comparison, an extensive matching process was carried out: For each navigated procedure performed, the institutional database was filtered for similar procedures performed under fluoroscopic guidance. Criteria were the number of levels instrumented (with deviations by one level considered acceptable), the area of the spine (upper thoracic, lower thoracic, thoracolumbar, lumbar, lumbosacral) and additional procedures performed (e. g., laminectomy). If several matches were available, the type of approach (percutaneous/open), the number of screws placed, indication for surgery, patient age and BMI were used in the order given as secondary criteria to find the best match. All procedures included in the fluoroscopy group were performed by spine surgeons with a similar level of experience as the surgeon who performed the procedures included in the navigation group.

In both groups, preoperative screw planning was performed using a DICOM viewer (IMPAX 6, Agfa Healthcare, Mortsel, Belgium) by measuring pedicle diameter and possible screw length in the computed tomography (CT) performed beforehand. In the fluoroscopy group, all screws were placed according to the preoperative screw planning. In the navigation group, intraoperative adjustments of the screw diameter and length were possible at the discretion of the surgeon according to the visualization of the screw in the correct trajectory.

For all procedures, patients were placed on a radiolucent carbon surgical table. In the navigation group, after preparation, draping and mounting of the patient array on a spinous process, a registration scan was performed using a latest generation mobile 3D C-arm cone beam computed tomography (CBCT, Cios Spin, Siemens Healthineers, Erlangen, Germany). During the 30 s scan acquisition, ventilation was paused resulting in higher quality of both imaging and navigation accuracy. The 3D datasets were then transferred to the Pulse platform (NuVasive, San Diego, CA, USA) for navigated pedicle screw placement. The implant systems used for dorsal stabilization in the navigation group were Reline MAS for percutaneous and Reline Open for open procedures (both NuVasive Inc., San Diego, CA, USA). The detailed screw placement technique with the navigation system used in this study has been published recently (Mandelka et al., 2022). An intraoperative 3D scan after screw placement to allow for control of implant position was only performed if the surgeon deemed it necessary as a result of limited assessability in 2D fluoroscopy.

In the fluoroscopy group, screw placement was performed under fluoroscopic guidance using a mobile 3D C-arm CBCT (Arcadis Orbic or Cios Spin, both Siemens Healthineers, Erlangen, Germany). Yamshidi needles were inserted into the pedicle under fluoroscopic control in both anteroposterior and lateral directions, then wires were passed through them, over which the screws were inserted. Systems used for dorsal stabilization in the fluoroscopy group were Reline MAS (33 cases), Reline Open (2 cases) or Precept (9 cases; all NuVasive Inc., San Diego, CA, USA). Exceptionally, in one of the cases included in the control group, the system Viper 2 (DePuy Synthes, West Chester, Pennsylvania, USA) was used. All systems used include cannulated polyaxial titanium screws, which were placed in the technique described above.

Screw accuracy was assessed in postoperative CT reconstructed in the axial, sagittal and coronal planes at average slice thickness of 2.0 mm by an independent experienced investigator according to Gertzbein-Robbins grading system (Gertzbein and Robbins, 1990). Grades A (no pedicle breach) and B (pedicle breach <2 mm) were considered acceptable, while Grades C to E (pedicle breach ≥2 mm) were considered potentially critical. The preoperative CT was used to measure pedicle width for the levels instrumented. Furthermore, the diameters of all screws were documented to calculate the screw/pedicle ratio.

Using the electronic patient chart and the dose report, the operating times (in minutes), intraoperative fluoroscopy time (in seconds) and intraoperative Dose Area Product (DAP, in mGycm (Rosinski et al., 2021)) were collected for all patients included in the study or the control group.

Statistical analysis was performed using Prism 9 (Graphpad Software, San Diego, CA, USA). Kolmogorov–Smirnov test was used to check for normal distribution of data. Descriptive statistics are shown as means ± standard deviation for continuous variables and absolute and relative numbers for nominal and ordinally scaled variables. For normally distributed continuously scaled data, a paired *t*-test was used to compare both groups, while Wilcoxon matched-pairs signed rank test was used as nonparametric alternative. For contingency testing, Fisher's exact test was used.

The mean screw pedicle ratio in both groups was compared using Mann-Whitney *U* test. The significance level was set at p < 0.05.

## 3 Results

A total of 45 patients with 379 screws, of which 361 were placed using 3D navigation, were included in the study group. The fluoroscopy group likewise consisted of 45 patients, with a total of 365 screws placed. A detailed comparison of patient and procedure characteristics for the two groups is displayed in Table 1.

**Table 1:** Comparison of the demographic data for both groups (BMI: body mass index).

		Navigation n = 45	Fluoroscopy n = 45	p		
Gender distribution [n]						
	Male [n (%]	21	(46.7)	31	(68.9)	0.054^a^
	Female [n (%]	24	(53.3)	14	(31.1)	
**Mean age [years]**	66.9 ± 14.8	57.9 ± 22.3	**0.043**^b^			
**BMI [kg/m**^**2**^**]**	27.6 ± 4.6	27.0 ± 5.6	0.589^x^			
**Surgical approach [n (%)]**						
	Percutaneous	24	(53.3)	26	(57.8)	0.832^a^
	Open	21	(46.7)	19	(42.2)	
**Indication for surgery [n (%)]**						
	Traumatic	27	(60.0)	34	(75.6)	0.250^a^
	Tumor/Pathologic fracture	8	(17.8)	5	(11.1)	0.550^a^
	Spondylodiscitis	9	(20.0)	4	(8.9)	0.230^a^
	Spondylolisthesis	1	(2.2)	2	(4.4)	>0.999^a^
**Number of segments fused [n (%)]**						
	1 segment	1	(2.2)	1	(2.2)	>0.999^a^
	2 segments	7	(15.6)	6	(13.3)	>0.999^a^
	3 segments	13	(28.9)	10	(22.2)	0.630^a^
	4 segments	16	(35.6)	19	(42.2)	0.666^a^
	5 segments	5	(11.1)	8	(17.8)	0.550^a^
	6 segments	3	(6.7)	1	(2.2)	0.616^a^
**Number of screws placed [n (%)]**						
	4 screws	2	(4.4)	2	(4.4)	>0.999^a^
	5 screws	1	(2.2)	0	(0.0)	>0.999^a^
	6 screws	6	(13.3)	6	(13.3)	>0.999^a^
	7 screws	0	(0.0)	1	(2.2)	>0.999^a^
	8 screws	23	(51.1)	24	(53.3)	>0.999^a^
	9 screws	1	(2.2)	1	(2.2)	>0.999^a^
	10 screws	4	(8.9)	9	(20.0)	0.230^a^
	11 screws	1	(2.2)	0	(0.0)	>0.999^a^
	12 screws	6	(13.3)	2	(4.4)	0.266^a^
	13 screws	0	(0.0)	0	(0.0)	>0.999^a^
	14 screws	1	(2.2)	0	(0.0)	>0.999^a^
**Additional surgical interventions [n (%)]**						
	None	22	(48.9)	26	(57.8)	0.526^a^
	Cement augmentation of screws	10	(22.2)	5	(11.1)	0.258^a^
	Laminectomy	12	(26.7)	14	(31.1)	0.816^a^
	Disk replacement	2	(4.4)	1	(2.2)	>0.999^a^
	Vertebroplasty/Kyphoplasty	3	(6.7)	0	(0.0)	0.242^a^
	Implant removal	2	(4.4)	0	(0.0)	0.494^a^
	Tumor debulking	0	(0.0)	1	(2.2)	>0.999^a^

a Fisher's exact test.

b Wilcoxon test.

x Paired *t*-test.

While the fluoroscopy group was significantly younger than the study group, there were no significant differences in gender distribution, body mass index (BMI) or the percentage of percutaneous and open procedures in the groups. There was also no significant difference in the indication for surgery, the number of fused segments, the number of screws placed, and the additional surgical interventions performed.

The intraoperative dose area product tended to be lower (p = 0.68), and the fluoroscopy time was significantly lower in the navigation group (p < 0.001; Table 2).

**Table 2:** Comparison of Dose Area Product, effective dose, fluoroscopy time in both groups.

	Navigation n = 45	Fluoroscopy n = 45	p
Dose Area Product [Gycm (Rosinski et al., 2021)]	24.18 ± 13.00	26.42 ± 19.40	0.680
Effective Dose [mSv]	6.19 ± 3.11	6.95 ± 4.97	0.431
Fluoroscopy time [s]	94.7 ± 29.4	210.0 ± 87.3	<0.001

The average operating time was 120.9 ± 47.3 min in the navigation group and 135.0 ± 65.1 min in the fluoroscopy group, showing no significant difference (p = 0.16).

In the navigation group, in 16 of the 45 cases included, an intraoperative 3D scan was performed after screw placement to allow for control of implant position. In two cases, the 3D scan resulted in revision of a single screw at levels T3 and L5.

**Fig. 1:**
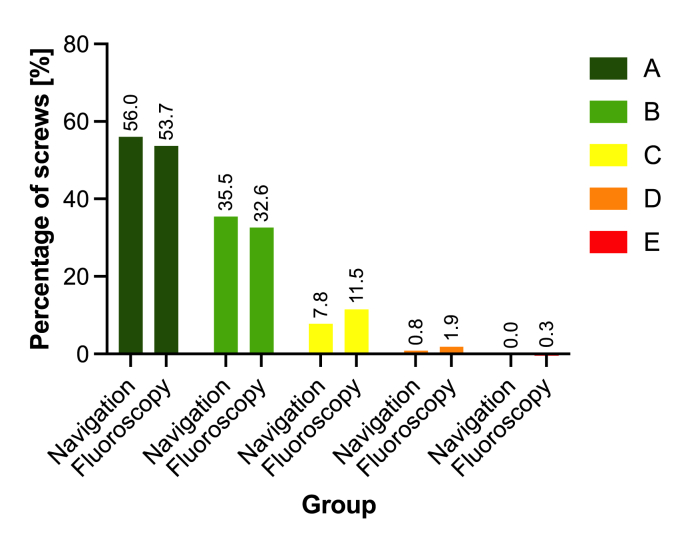
Comparison of screw accuracy according to Gertzbein-Robbins classification for both groups.

**Fig. 2:**
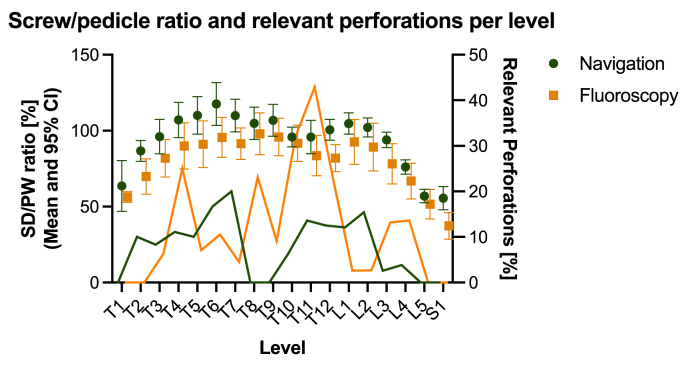
Comparison of the SD/PW ratio and the rate of relevant perforations per level for both groups.

The final screw accuracy as measured in postoperative computed tomography was 91.4% in the navigation group and 86.3% in the fluoroscopy group (p = 0.034; Fig. 1). While the screw accuracy was significantly higher in percutaneous procedures with navigation (90.7%) than in such without navigation (82.6%; p = 0.025), no significant difference was found for open procedures with an accuracy of 92.2% using navigation and 90.9% with fluoroscopic assistance (p = 0.430). The rate of relevant pedicle perforations per level is shown in Fig. 2.

In the navigation group, 28 grade C perforations (7.8%) and 3 grade D perforations (0.8%) were found. Except for 2 grade C perforations at levels (at levels T2 and T8), all screws with relevant perforations occurred on the lateral side of the pedicle. All but one screw with grade C or grade D perforations showed a so-called ‘in-out-in configuration’. Relevant pedicle breaches on levels T4 to T7 and T11 to L2 accounted for 80.6% of all grade C and D breaches.

The assessment of screw accuracy in the fluoroscopy group found 50 screws with pedicle breaches corresponding to grade C to grade E. Of these screws, four screws showed a medial breach (2 grade C at levels T4 and T8, 1 grade D at level L2 and 1 grade E at level L2) and 46 screws showed a lateral perforation (40 grade C and 6 grade D). Of the 50 screws, 42 showed an ‘in-out-in configuration’ while 8 screws showed a parapedicular and paravertebral screw course. In the case with the exceptional use of a different implant system, the four screws placed were assessed as Grade A.

None of the pedicle breaches reported in the present study resulted in neurological deficits, accordingly, no revision surgery due to screw misplacement was performed.

In the navigation group, postoperative complications were seen in five patients (11.1%), of which four were cases with open surgery: Two cases with non-infected wound-healing disorders (4.4%), two cases with pull-out of a single screw each on levels T11 and L2 in osteoporotic patients despite primary cement augmentation (4.4%) and one case with postoperative hematoma (2.2%). In all five cases, revision surgery was performed. In the pullout-cases, extended re-instrumentation was performed.

In the fluoroscopy group, postoperative complications were seen in two patients (4.4%), of which one had had open surgery. Both patients showed postoperative hematoma for which revision surgery was performed. There was no significant difference regarding the occurrence of postoperative complications between the groups (p = 0.434).

In the navigation group and the fluoroscopy group, all complications occurred within six weeks after the initial surgical procedure. Except for implant removal, after a mean follow-up time of 16 ± 3 months in the navigation group and 27 ± 9 months in the fluoroscopy group, there were no additional re-admissions for further complications.

**Fig. 3:**
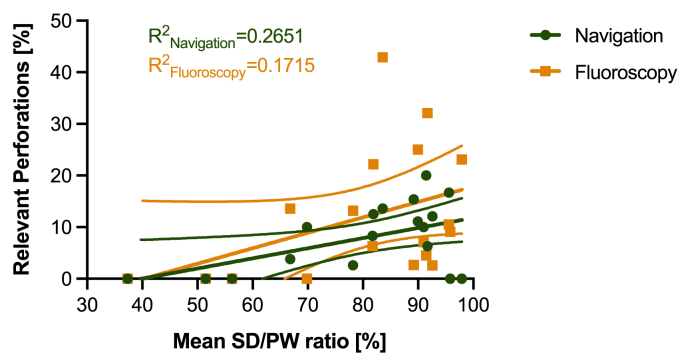
Correlation of relevant pedicle perforation rate with SD/PW ratio (mean ± 95% confidence interval) for spine levels T1 to S1.

Taking into account the individual screw diameter and pedicle diameter for each screw, there was a significantly greater SD/PW ratio in the navigation group compared with the fluoroscopy group (95.8 ± 24.3% vs. 85.1 ± 17.8%, p < 0.0001; Fig. 2). Interestingly, the mean pedicle width was significantly smaller in the navigation group (7.1 ± 2.9 mm vs. 8.0 ± 3.0 mm, p < 0.0001). For both groups, we found a weak correlation (R^2^) between the mean SD/PW ratio and the rate of relevant perforations (Fig. 3).

## 4 Discussion

Our results, in accordance with previous studies, show a higher accuracy for navigated compared to fluoroscopic-guided screw placement, especially for minimally invasive procedures. This is even more significant as the SD/PW ratio, and thus the risk of perforation, was significantly greater in the navigated group. The fact that the average pedicle width was significantly smaller in patients who underwent surgery with navigation guidance further suggests adequate case selection for the use of intraoperative navigation at our institution.

In our approach, higher accuracy is not achieved at the expense of higher intraoperative radiation exposure for the patient. On the contrary, the radiation dose even tended to be lower in the navigation group. While the comparability of these results with other studies is limited by the fact that these results depend on the individual surgical technique and, in particular, the frequency of use of intraoperative 2D and 3D imaging, Tkatschenko et al. concluded that a potentially higher one-time radiation exposure may be outweighed by the benefits of three-dimensional (3D) navigation (Tkatschenko et al., 2020). Even more important is the fact that navigation has been reported to reduce radiation exposure for surgical personnel, who are exposed to a substantial amount of radiation exposure throughout the years, particularly in spine surgery. Anecdotally, this is consistent with our experience. A study by Villard et al. found that navigation reduced the surgeon's radiation exposure by a factor of 10 compared to fluoroscopically assisted pedicle screw placement due to minimized use of fluoroscopy and reduced exposure of surgical personnel during scan acquisition (Villard et al., 2014). In our study population, the use of navigation also did not increase the operating time, in fact, procedures even tended to be slightly shorter in the navigation group.

Numerous studies have investigated different factors that influence the biomechanical properties of screws used for spinal fixation. The size, design, entry point and the trajectory of the screw as well as bone mineral density (BDM) have been named relevant factors (Bianco et al., 2017; Shea et al., 2014; Matsukawa et al., 2021; Alhabib et al., 2011; Chatzistergos et al., 2010; Cho et al., 2010; Cook et al., 2004; Hirano et al., 1997; Hsu et al., 2005; Karami et al., 2015; Krag et al., 1988; Lehman et al., 2003; Paxinos et al., 2010; Santoni et al., 2009; Takeshita et al., 2009; Weinstein et al., 1992). When determining the screw trajectory, there is a distinction between the so-called anatomical technique along the anatomical pedicle axis and the so-called straightforward technique. While the former allows the use of longer screws with a larger diameter, the latter aims to place the screws closer to the cortical bone to improve the stability of the screw. At the same time, however, there is a higher risk of pedicle perforation with this technique (Bianco et al., 2017; Santoni et al., 2009). In our institution, the anatomical technique is used. Yet, whenever possible, the cortical layer grip to both the medial and the lateral cortex is maximized by using the largest screw diameter possible which explains the high SD/PW ratio seen for the thoracic spine in our study.

The increase in screw diameter has been identified as the most important factor in achieving higher pullout strength (Bianco et al., 2017; Cho et al., 2010; Helgeson et al., 2013) The use of screws of a larger diameter leads to compression of the surrounding subcortical bone mass, which enlarges the contact area of the screw and thus distributes the load of the screw over a larger area (Bianco et al., 2017; Matsukawa et al., 2021; Hsu et al., 2005).

In their meta-analysis, Solitro et al. listed the recommendations of various studies regarding the suggested screw diameters, showing considerable differences. While some authors advocated the so-called “pedicle fit and fill” theory (Lehman et al., 2003), others promoted a fixed ratio of screw diameter to pedicle width (SD/PW ratio). The recommended SD/PW ratio ranged from 80 to 125% (Solitro et al., 2022; Suk et al., 2001; Di Silvestre et al., 2007; Gstoettner et al., 2011). Other authors recommended that the screw diameter should be 1 mm larger than the minimum endosteal diameter of the pedicle (Misenhimer et al., 1989; Matsukawa et al., 2022; Li et al., 2004). Ultimately, the authors came to the conclusion that pedicle perforation rates were lowest at an SD/PW ratio of 0.8, so this ratio was considered as a recommended value for inexperienced surgeons (Solitro et al., 2019). In contrast, using highly precise navigated techniques, screw selection at the upper limit of the range recommended above seems to be possible.

Due to the lack of proper guidelines or standards, many spine surgeons choose the appropriate screw size based on their experience or based on patient size, which may in some cases lead to the selection of screws that are significantly too small or too large (Bianco et al., 2017; Matsukawa et al., 2015, 2022; Kosmopoulos and Schizas, 2007). While this leads to lower fixation strength in the former case, screws that are too larg e can cause pedicle perforations and, in the worst case, neurological or vascular complications (Helgeson et al., 2013). Another risk of oversizing screws is fracturing of the pedicle, which according to a study by George et al. reduces the pullout strength by 11% compared to the intact pedicle (George et al., 1991). Despite the high SD/PW ratio in our study, the rate of relevant pedicle perforations remains acceptable, and no evidence of pedicle fractures was found in postoperative computed tomography. Accordingly, our results highlight that aiming for maximal SD/PW ratio is not necessarily associated with a higher rate of relevant pedicle perforations or pedicle fractures.

Still, in retrospect, the limits may have been pushed with some of the screws placed in the navigation group, especially in the thoracic spine. In a trial by Korkmaz et al. it was shown that the pullout strength without pedicle perforation was 70% higher than with a high-grade lateral pedicle perforation. Correction of an initially laterally perforating screw with subsequent complete intrapedicular placement was shown to be biomechanically significantly advantageous, as a large portion of the lost pullout strength is regained (Korkmaz et al., 2018). Ye et al. also investigated the biomechanical effects of correcting the screw trajectory after initial misplacement. The authors concluded that repositioning of screws is biomechanically reasonable only if the overlap between old and new trajectory is smaller than 40% (Ye et al., 1976).

Matsukawa et al. demonstrated in their study that screw diameter had a greater effect on resistance to screw pullout and flexion-extension loading, while greater screw length, commonly measured as %depth of the vertebral body, proved to be advantageous in lateral bending and axial rotation loading (Matsukawa et al., 2021; Krag et al., 1988; Bezer et al., 2012). Liu et al. found significant differences in terms of functional and radiological outcome in patients with longer (80%depth) compared with shorter screws (<60%depth) (Liu et al., 2020). Weinstein et al. reported that the pedicle contributes 60% to the pullout strength of pedicle screws, while only 15–20% is accounted for by the lower-density cancellous bone of the vertebral body. 20–25% higher stability could be achieved by penetrating the anterior vertebral cortex (Hirano et al., 1997; Karami et al., 2015; Weinstein et al., 1992), however, this should be avoided due to the small distance of 2.4–3.2 mm to the prevertebral vessels in the lumbar spine (Kot et al., 2022). Following their radiological analysis, Chua et al. recommended that screws should be selected no longer than 75–80% of the vertebral body depth to avoid complications caused by anterior perforation (Chua et al., 2019).

Overall, in screw planning, spine surgeons have to deal with the trade-off between avoiding relevant perforations while maximizing screw diameter and length as well as the amount of cortical layer grip. Unfortunately, the literature suggests that even the use of preoperative computed tomography to determine screw diameters may also lead to inadequate screw selection (Helgeson et al., 2013; Matsukawa et al., 2022). When comparing pedicle diameters of the lumbar spine in the axial plane and actual pedicle diameters using multiplanar reconstructions, Makino et al. demonstrated that the actual diameter in the axial plane was frequently overestimated (Makino et al., 2012). Accordingly, Matsukawa et al. conclude that the screw size should be individually adapted to the pedicle diameter and the possible screw insertion depth into the vertebral body (Matsukawa et al., 2022). This procedure corresponds to the workflow we perform on a daily basis with navigated pedicle screw placement, where different screw sizes can be previsualized followed by adequate screw selection. Still, the evidence in the literature regarding implications of the use of navigation on the screw size is very limited (Du et al., 2022).

Our results show that even in a small sample, navigated pedicle screw placement has advantages in terms of a significantly higher SD/PW ratio with the corresponding biomechanical implications for dorsal stabilization, while ensuring the high accuracy of screw placement especially in minimally invasive screw placement. Recent studies suggest that the use of robotic-assisted techniques could potentially optimize these results even further (Kanaly et al., 2022; Shafi et al., 2022).

The results of the present study should be interpreted within the confines of study limitations. Due to the study being a review of single-surgeon, single-center data with a limited number of cases and little clinical follow-up data, the generalizability of the results is limited. However, an extensive matching process was performed, and the results of the navigation group were compared to similar fluoroscopy-guided procedures performed in our institution. Furthermore, the data available in the literature support the results presented above. Future studies should investigate whether the increase in SD/PW ratio enabled by navigation and/or robotic assistance contributes to a better clinical outcome.

## 5 Conclusions

In conclusion, this study was able to show that navigated thoracolumbar pedicle screw placement not only increases the accuracy of screw placement but also facilitates the selection of the adequate screw sizes, which according to the literature has positive effects on fixation strength. Meanwhile, the use of navigation did not negatively affect the time needed for surgery or the patient's intraoperative exposure to radiation.

## Contributions

Conception and design of the study: PAG, JF, SYV. Data Acquisition: EM, JG, FZ. Analysis and Interpretation of Data: EM, SYV. Writing of the manuscript: EM, JG. Revision of the manuscript: FZ, PAG, JF, SYV. All authors have read and approved the final version of the manuscript.

## Funding

This research did not receive any specific grant from funding agencies in the public, commercial, or not-for-profit sectors.

## IRB approval

The study was reviewed and approved by the responsible Ethics Committee (application number 2021–16061). All patients provided verbal and written consent. All procedures were performed in accordance with the ethical standards of the institutional and/or national research committee and the 1964 Helsinki Declaration and its later amendments or comparable ethical standards.

## Data availability

All data and statistics are available on request from the corresponding author.

## Declaration of competing interest

PAG reports a relationship with Siemens Healthineers that includes: consulting or advisory and travel reimbursement. JF reports a relationship with Siemens Healthineers that includes: consulting or advisory and travel reimbursement.

The research group received grants/has grants pending and technical support from Siemens Healthineers (Erlangen, Germany) and Nuvasive Inc. (San Diego, CA, USA). The funders had no involvement in the study design, collection, analysis, and interpretation of data, writing of the manuscript, or decision to submit the manuscript for publication.
